# A SARS-Cov-2 sensor based on upconversion nanoparticles and graphene oxide†

**DOI:** 10.1039/d2ra03599e

**Published:** 2022-06-22

**Authors:** Konstantina Alexaki, Maria Eleni Kyriazi, Joshua Greening, Lapatrada Taemaitree, Afaf H. El-Sagheer, Tom Brown, Xunli Zhang, Otto L. Muskens, Antonios G. Kanaras

**Affiliations:** School of Physics and Astronomy, Faculty of Engineering and Physical Sciences, University of Southampton Southampton SO17 1BJ UK a.kanaras@soton.ac.uk; College of Engineering and Technology, American University of the Middle East Kuwait; Department of Chemistry, University of Oxford, Chemistry Research Laboratory 12 Mansfield Road Oxford OX1 3TA UK; Chemistry Branch, Department of Science and Mathematics, Faculty of Petroleum and Mining Engineering, Suez University Suez 43721 Egypt; School of Engineering, Faculty of Engineering and Physical Sciences, University of Southampton Southampton SO17 1BJ UK; Institute for Life Sciences, University of Southampton Southampton SO171BJ UK

## Abstract

Since the beginning of the COVID-19 pandemic, there has been an increased need for the development of novel diagnostic solutions that can accurately and rapidly detect SARS-CoV-2 infection. In this work, we demonstrate the targeting of viral oligonucleotide markers within minutes without the requirement of a polymerase chain reaction (PCR) amplification step *via* the use of oligonucleotide-coated upconversion nanoparticles (UCNPs) and graphene oxide (GO).

## Abstract 2

A simple and sensitive sensor made of upconversion nanoparticles and graphene oxide to detect SARS-CoV-2 RNA rapidly.

## Graphical abstract

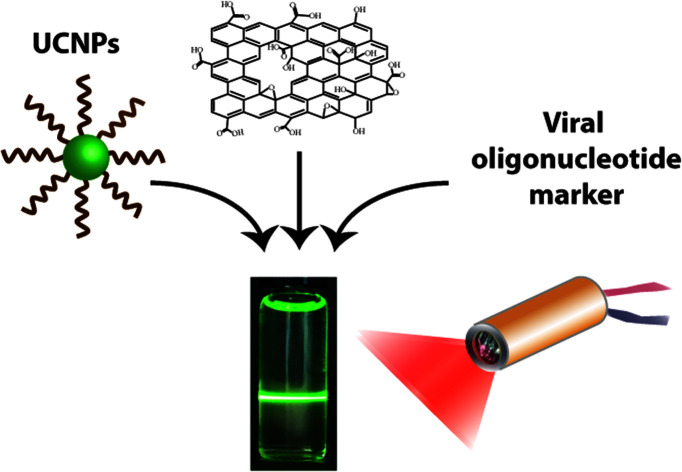

In 2019 a novel form of coronavirus was identified termed severe acute respiratory syndrome coronavirus 2. The disease, also known as COVID-19, was found to be highly transmissible amongst humans and in some cases fatal. This led to a global urgent requirement for the development of sensitive and specific diagnostic tools for the detection of the virus and its subsequent mutated versions.

The most reliable technique in clinical diagnosis relies on the detection of the RNA of SARS-CoV-2 using nucleic acid amplification technologies (NAATs) and primarily real-time polymerase chain reaction (RT-PCR).^1^ This technique, although reliable, is hindered by the need for specialized equipment and long turnaround times. To address these limitations alternative diagnostic tools have recently been developed based on the use of biosensors. A biosensor platform typically consists of three important features, a bio-receptor such as a protein, a transducer component, and an amplification element. Most commonly the bio-receptor interacts with the biomarker of interest and induces a change, which depending on the transducer could be observed as an electrochemical, optical, electrical, or thermal change thus signalling the presence of the target.^2–4^ These sensors represent a testing method that is sensitive and thus have recently been used for the detection of SARS-CoV-2 virus markers in order to control and diagnose COVID 19.^2,5^ For example, Seo and co-workers reported the development of a field-effect transistor (FET) based biosensor for the detection of the SARS-CoV-2 spike protein. Their device was found to be highly sensitive towards the target and exhibited no cross-reactivity with the MERS-CoV-2 virus.^6^ Furthermore, Lin *et al.* demonstrated a rapid and sensitive lateral flow immunoassay (LFIA) which consisted of lanthanide-doped polystyrene nanoparticles for the detection of anti-SARS-CoV-2 IgG in human serum.^7^ Moitra and co-workers focused on the detection of viral RNA and developed a colorimetric assay based on the use of gold nanoparticles (AuNPs) functionalized with thiol-modified antisense oligonucleotides (ASOs) specific to the N-gene of SARS-CoV-2. In the presence of the target RNA, selective AuNP agglomeration was observed as indicated by the change in the surface plasmon resonance (SPR) of the AuNPs. The phosphodiester bonds of the SARS-CoV-2 RNA (N gene) strand were subsequently cleaved following the addition of RNaseH leading to a visually detectable precipitate mediated by the additional agglomeration of AuNPs. By testing against alternative RNA targets they confirmed not only the sensitivity but also the specificity of their method.^8^ Fluorescence based detection was shown by Wang *et al.* who developed an immunoassay, implemented on a lateral flow strip for the detection of SARS-CoV-2 RNA. They made use of S9.6 monoclonal antibody-labelled europium-chelate-based fluorescent nanoparticles (FNP) capable of capturing double strands. Within 1 h they were able to detect the absence or presence of the RNA target *via* the use of a fluorescence analysis device with high sensitivity and specificity.^9^

When looking at the use of alternative sensors for the detection of COVID-19 there are some important objectives that should be taken into consideration. First the sensor should be highly selective and specific, which means that it should bind rapidly and accurately only to the target molecules. Second, the sensor must detect the target molecule to the smallest possible amounts to enable early diagnosis (*e.g.* important for controlling the spread of a disease). Third the sensor should be easy and cheap to fabricate in large scales so that it can be easily commercialized. Furthermore, it needs to be stable in a range of conditions (*e.g.* temperature variations during transportation) as well as being made by non-toxic materials to avoid contamination of the environment.

An interesting oligonucleotide sensor, which we have recently developed, and takes into consideration the aforementioned points, involves the use of UCNPs and GO. This sensor uses a co-doping system of NaYF_4_:Yb^3+^, Er^3+^ UCNPs, which comprises of three components: a host matrix (NaYF_4_), sensitiser (Yb^3+^) and activator (Er^3+^).^10^ One important advantage involving the use of UCNPs is the ability to easily modify their surface with synthetic oligonucleotides. In the presence of GO, ‘π–π stacking’ intermolecular interactions take place between the oligonucleotide aromatic bases and the sp^2^ hybridised carbon structure of GO, leading to adsorption of the UCNPs on the GO surface and complete fluorescence quenching of the system.^11^ Previous research has suggested that when UCNPs and GO are used as a donor/acceptor pair, a FRET quenching mechanism is adopted. The energy transfer is based on donor–acceptor dipole–dipole interactions and depends on the degree of overlap between absorption and emission spectra as well as the donor–acceptor distance, which should be less than 10 nm.^11–15^ In the presence of the complementary oligonucleotide target, preferential hydrogen bond hybridisation of bases occurs and UCNPs can no longer adsorb to the GO surface. Fluorescence quenching therefore no longer occurs and a fluorescence signal is recorded.^16^ We have previously shown how such a biosensor could successfully be used for the detection of Alzheimer's and prostate cancer mRNA markers in the presence of complex biological environments such as human blood serum and cell lysate.^17^ We also demonstrated the construction of a portable sensor and its use in the field for the detection of mRNA biomarkers related to Zn deficiency in crops.^18^

In this paper, we demonstrate the construction of an UCNP/GO biosensor for the sensitive and rapid detection of oligonucleotide markers associated with the SARS-CoV-2 virus as shown in Scheme 1. UCNPs were functionalized with oligonucleotides designed to detect part of the viral genome coding for the RNA-dependent RNA polymerase (RdRp)/Helicase (Hel), a marker that is also used in RT-PCR testing.^19^ We show that the presence of the target can be detected within a few minutes with excellent selectivity and sensitivity.

**Scheme 1:**
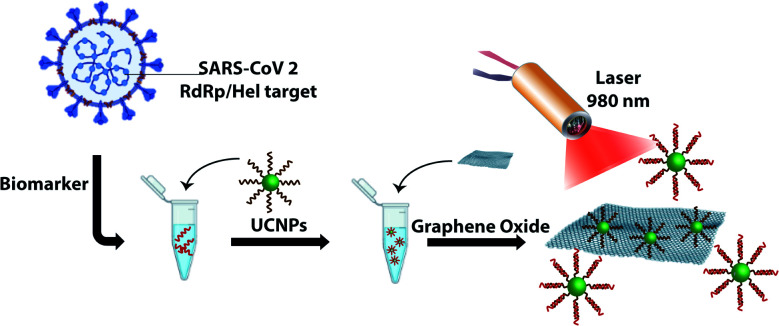
Detection of an oligonucleotide target associated with the RdRp/Hel gene of SARS-CoV-2. Oligonucleotide-coated UCNPs designed to detect part of the SARS-CoV-2 gene are incubated with the target of interest. Upon addition of GO and irradiation with a laser at 980 nm, the fluorescence signature originating from the UCNPs is monitored. The absence of a fluorescence signal would indicate that the target is not present and fluorescence quenching has taken place following adsorption of the nanoparticles onto the GO surface.

A solvothermal method was used for the synthesis of hexagonal phase β-NaYF_4_:Yb^3+^ (20%), Er^3+^ (2%) upconversion nanoparticles (core UCNPs).^18,20^Fig. 1 shows the resulting core UCNPs, which were highly monodisperse with an average size of 33 ± 1 nm (Fig. 1A and C). Core UCNPs were further coated with an additional shell of NaYF_4_, which has been proven to enhance the upconversion fluorescence emission as presented in Fig. S1.†^21^ The shell was achieved by following a slightly modified version of a previously reported method.^18^ The resulting core–shell UCNPs retained their uniform shape and had a larger average size of 37 ± 1 nm (Fig. 1B and D) due to the shell formation.

**Fig. 1:**
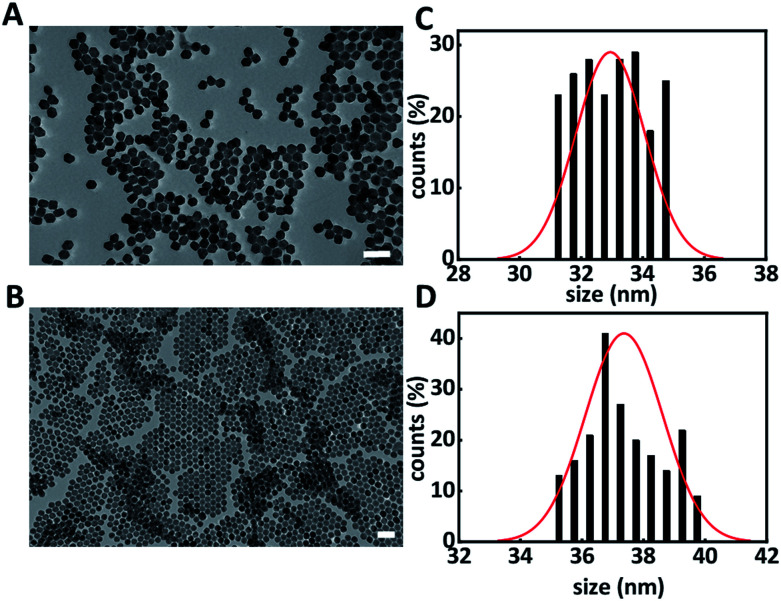
Representative transmission electron microscopy images of core (A) and core–shell UCNPs (B) and their corresponding size distribution histograms (C and D). Scale bar is 100 nm.

The synthesised nanoparticles demonstrated the expected fluorescence signal corresponding to ytterbium and erbium doped NaYF_4_ nanoparticles following excitation with a 980 nm laser. The fluorescence spectrum demonstrated two distinct peaks at 540 nm and 655 nm (see Fig. S1†) where the green emission peak at 540 nm corresponded to the ^4^S_3/2_ → ^4^I_15/2_ transition and the red emission peak at 655 nm corresponded to the ^4^F_9/2_ → ^4^I_15/2_ transition.^22^ Following their synthesis, UCNPs were well dispersed in hexane and a number of surface modification steps were subsequently followed in order to achieve water solubility and the covalent attachment of single-stranded DNA (ssDNA) sequences on the UCNP surface.

Following the successful multi-step synthesis and characterisation of single-stranded oligonucleotide coated UCNPs (ssDNA-UCNPs), we evaluated the ability of GO to quench UCNP fluorescence by performing a calibration experiment. This was aimed to determine the optimum UCNP/GO ratio for complete UCNP fluorescence quenching. Fig. 2A shows that by gradually increasing the concentration of GO, whilst maintaining the same concentration of UCNPs (0.5 mg mL^−1^) a steady decrease in the fluorescence intensity was observed with maximum quenching observed at a GO concentration of 0.6 mg mL^−1^.

**Fig. 2:**
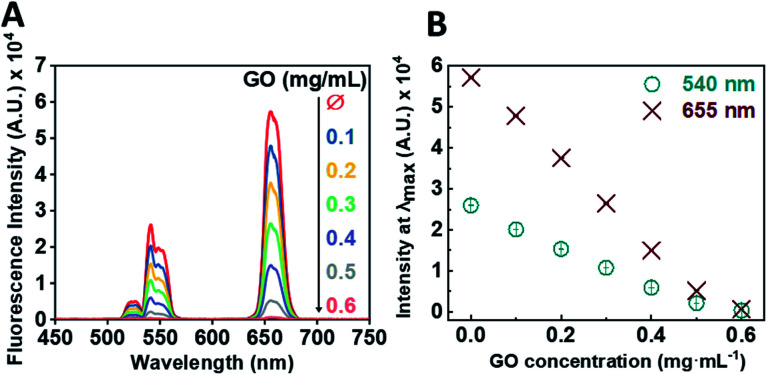
(A) Representative fluorescence emission spectrum from oligonucleotide coated UCNPs (0.5 mg mL^−1^) in the presence of increasing concentrations of GO. (B) Correlated fluorescence emission spectrum from oligonucleotide coated UCNPs (0.5 mg mL^−1^) showing the decreasing fluorescence emission of the *λ*_max_ of the two typical peaks of UCNPs (655 nm, red points; 540 nm, cyan points) in the presence of increasing concentration of GO.

We further correlated the change in fluorescent intensity at *λ*_max_ for each UCNP peak upon the addition of GO. Fig. 2B shows the change in fluorescence intensity at wavelengths of 540 nm and 655 nm as a function of GO concentration. Upon the addition of 0.6 mg mL^−1^ of GO to the oligonucleotide coated UCNP solution, the fluorescence signal was almost completely quenched.

SARS-CoV-2 is a positive strand RNA virus with a genome that is approximately 30 kb in size. One core component of the virus genome is known as RNA dependant RNA polymerase (RdRp) and is critical for the replication and transcription of viral RNA.^23^ Amongst other targets, the detection of RdRp *via* RT-PCR has been reported to have the highest analytical sensitivity and it was chosen as a target for our sensor.^19^

Following calibration experiments, we proceeded to determine whether our sensor could efficiently detect the presence of the RdRp target. In the absence of a target and upon incubation with GO (0.6 mg mL^−1^), no fluorescence signal could be detected. For the target experiment, the ssDNA-UCNPs were hybridized to their complementary DNA (cDNA) (see Table S1†) and then GO was added. Fig. 3 presents the results obtained after hybridisation with an increasing concentration of cDNA target for 30 min. A decrease in the quenching efficiency of GO (Fig. 3A) over both characteristic peaks was observed when the concentration of the target was increased from 5 fM to 50 nM. The formation of double-stranded DNA UCNPs (dsDNA-UCNPs) prevented their adsorption onto the GO surface and their fluorescence was retained. Fig. 3B shows the maximum intensity recorded for the two characteristic UCNP peaks at 540 nm and 655 nm as a function of cDNA concentration. It was observed that at lower concentrations of cDNA the fluorescence intensity was weak. Most likely, this was due to a low number of duplexes present on the UCNPs which did not prevent some particles to adsorb to the GO surface. As the target concentration increased, the number of DNA duplexes on the nanoparticles also increased preventing the adsorption of nanoparticles to the GO surface and giving a strong fluorescent signal coming from the UCNPs. We further investigated the functionality of our sensor within biological fluids (see Fig. S5†) or by the use of a complementary RNA (cRNA) target (see Fig. S6†) as opposed to a cDNA target. In both instances our sensor demonstrated a rapid response to the complementary target.

**Fig. 3:**
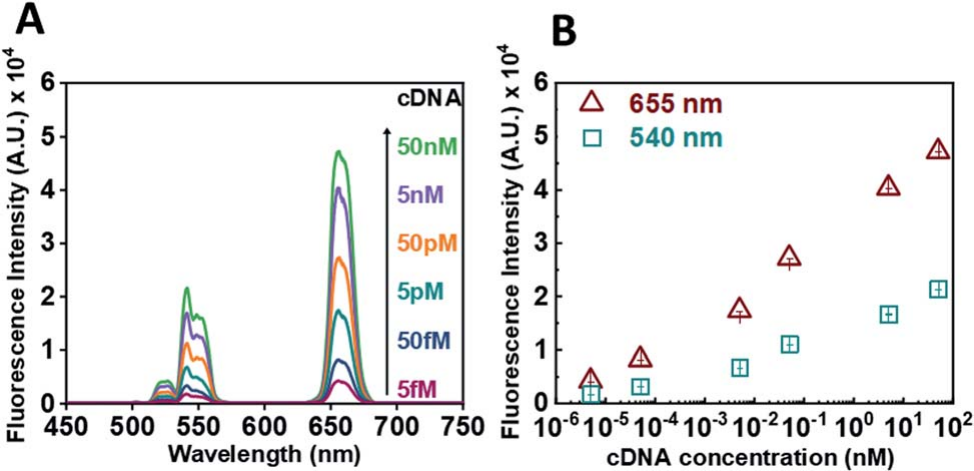
(A) Representative fluorescence spectrum of oligonucleotide coated UNCPs (0.5 mg mL^−1^) after incubation with increasing concentrations of cDNA targets in the presence of GO (B) graph of the maximum UCNP fluorescence intensity measured at 540 nm (cyan points) and 655 nm (red points) in the presence of GO as a function of cDNA concentration.

The specificity of the sensor was further investigated to determine whether accurate detection could be ensured. Fig. 4 shows that upon incubation with a non-complementary DNA (ncDNA) target followed by the addition of GO (0.6 mg mL^−1^) no fluorescence signal could be detected. As the concentration of the non-complementary target was progressively increased from 5 nM to 1000 nM the fluorescence signal remained quenched. This indicated that no duplex formation took place thus allowing ssDNA-UCNPs to adsorb to the GO surface leading to fluorescence quenching.

**Fig. 4:**
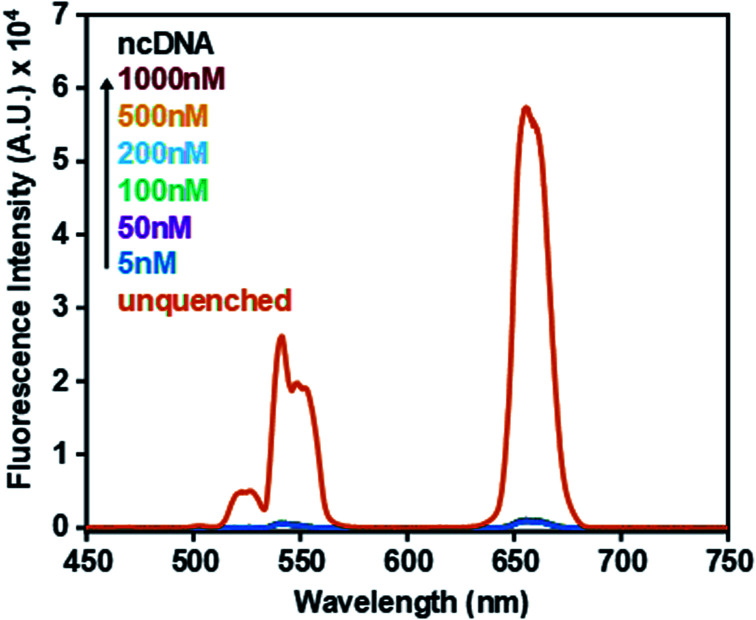
Fluorescence spectrum of oligonucleotide coated UCNPs (0.5 mg mL^−1^) in the absence of ncDNA and GO (unquenched) and in the presence of increasing concentrations of a ncDNA sequence and GO (0.6 mg mL^−1^). For comparison the fluorescence spectrum of oligonucleotide coated UCNPs in the absence of GO is shown.

The lowest detected concentration in our experiments was 5 fM. This concentration was measured in bulk solution without concentration enrichment. The optical pumping was done using a focused pencil beam of around 20 × 20 × 500 μm^3^ dimensions, which excited UCNPs in a volume of 0.2 nL. At the concentration of 5 fM we therefore estimate a total number of 0.6 target copies in the detection volume at any given time. Furthermore, implementations might benefit from schemes in which further concentration of analytes is achieved through enrichment, ideally combined with a simple lateral flow configuration.^9,24,25^

In conclusion, we have demonstrated the construction of a sensor for the detection of a viral oligonucleotide target, a fingerprint of the SARS-CoV-2 virus with high sensitivity and specificity.

## Conflicts of interest

There are no conflicts to declare.

## Supplementary Material

RA-012-D2RA03599E-s001
