# Supplementary material for: A SARS-Cov-2 sensor based on upconversion nanoparticles and graphene oxide
Source: RSC Adv. 2022 Jun 22;12(29):18445–9. doi: 10.1039/d2ra03599e (PMC9215703; doi:10.1039/d2ra03599e)
Supplement: RA-012-D2RA03599E-s001 [file RA-012-D2RA03599E-s001.pdf]

Electronic Supplementary Material (ESI) for RSC Advances.
This journal is © The Royal Society of Chemistry 2022

Supplementary Information

A SARS-CoV-2 Sensor Based on Upconversion Nanoparticles and Graphene Oxide

Konstantina Alexaki,[a] *Maria-Eleni Kyriazi,*[b] *Joshua Greening,*[a] *Lapatrada Taemaitree,*[c] *Afaf H. El-Sagheer,*[c,d] *Tom Brown,*[c] *Xunli Zhang,*[e,f] *Otto L. Muskens,*[a,f] *Antonios G. Kanaras*[a,f,*]

[a] School of Physics and Astronomy, Faculty of Physical Sciences and Engineering, University of Southampton, Southampton SO171BJ, United Kingdom.

[b] College of Engineering and Technology, American University of the Middle East, Kuwait

[c] Department of Chemistry, University of Oxford, Chemistry Research Laboratory, 12 Mansfield Road, Oxford OX1 3TA, UK.

[d] Chemistry Branch, Department of Science and Mathematics, Faculty of Petroleum and Mining Engineering, Suez University, Suez 43721, Egypt.

[e] School of Engineering, Faculty of Physical Sciences and Engineering, University of Southampton, Southampton SO171BJ, United Kingdom.

[f] Institute for Life Science, University of Southampton, Southampton, SO171BJ, United Kingdom.

Table of Contents

S - I **Characterization of UCNPs**

S - II **Characterization of PAA-UCNPs and oligonucleotide UCNPs**

S - III **Oligonucleotide synthesis and purification**

S-IV **UV-Vis characterization of GO**

S-V **Target recognition in biological fluid**

S-VI **RNA target recognition**

S-VII **Additional experimental information**

S-I Characterization of UCNPs

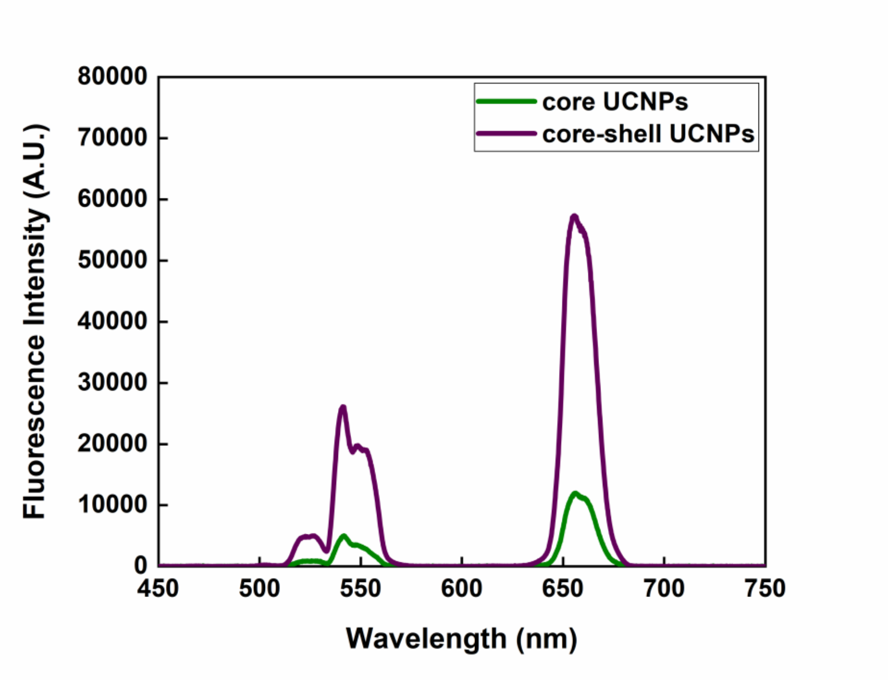

Figure S1. Fluorescence emission spectra of core UCNPs (green line) and core-shell UCNPs (purple line) in hexane irradiated with a 980 nm laser beam. The peak positions remained the same since the percentage of erbium and ytterbium was constant in both samples.

S-II Characterization of PAA-UCNPs and oligonucleotide UCNPs

Following the solvothermal synthesis, core-shell UCNPs had oleic acid (OA) ligands on their surface. Initially, a ligand exchange procedure was performed where OA ligands were replaced with poly-acrylic acid (PAA). This enabled the transfer of UCNPs to water and facilitated their subsequent functionalization with amino-modified oligonucleotides. The chemical coupling was performed utilizing an EDC coupling reaction to create an amide bond between the amino groups of oligonucleotides and the carboxylic groups of the PAA on the UCNP surface. Successful coupling was assessed *via* zeta potential (see **Figure S2**) and Fourier transform infrared spectroscopy (FTIR) (see **Figure S3**). A clear decrease in the net charge was observed upon oligonucleotide functionalization, which was attributed to the increase in negative charge arising from the oligonucleotide backbone. FTIR analysis showed that upon oligonucleotide coupling the characteristic peak corresponding to the carboxyl group of PAA disappeared and two new peaks at1650 cm^{-1} and 1560 cm^{-1} corresponded to the vibrations of the amide group (C=O and N-H) appeared.

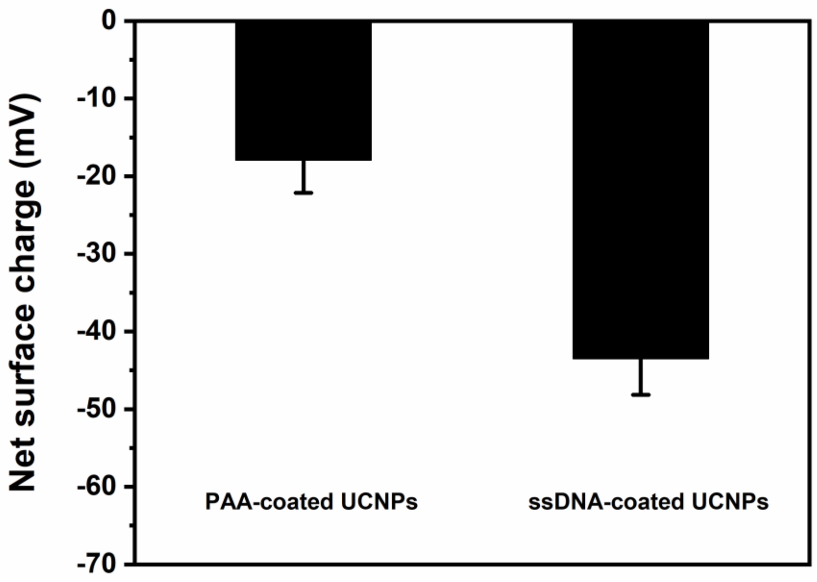

Figure S2. Zeta potential measurements of PAA coated core-shell UCNPs and oligonucleotide coated core-shell UCNPs (0.5 mg/mL). A clear change in the zeta potential charge of the nanoparticles was observed indicating successful oligonucleotide coupling.

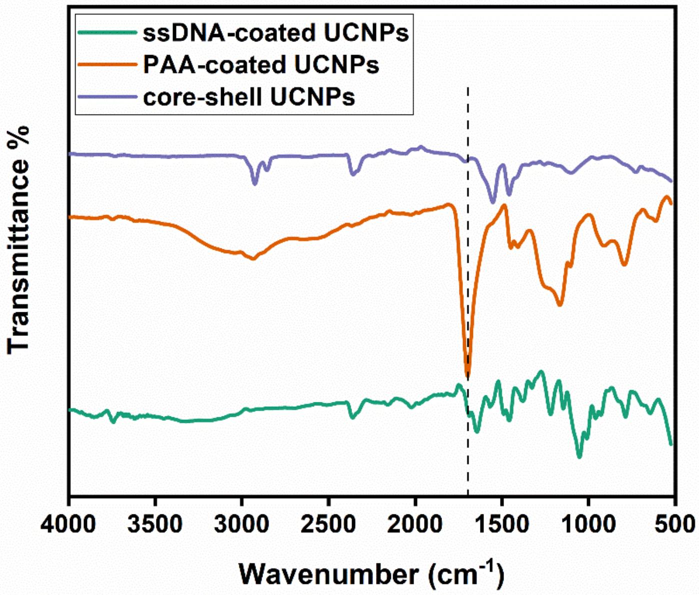

Figure S3. Fourier-transform infrared spectroscopic measurements of oleate-capped core-shell UCNPs, PAA coated core-shell UCNPs and oligonucleotide coated core-shell UCNPs. The presence of PAA is confirmed by

the appearance of a strong COOH peak at 1700 cm^{-1}. The appearance of the peaks at 1650 cm^{-1} and 1560 cm^{-1} is related to the creation of amide bonds due to the conjugation of the oligonucleotides *via* EDC coupling.

Table S1: Oligonucleotide sequences. X: aminohexyl modification

**Name**	**Oligonucleotide sequences (5' to 3') and modifications**
**COVID-19 RdRP/Hel (cDNA target sequence)**	TTAAGATGTGGTGCTTGCATACGTAGAC
**COVID-19 RdRP/Hel (cRNA target sequence)**	UUAAGAUGUGGUGCUUGCAUACGUAGAC
**COVID-19 RdRP/Hel (sense strand)**	X-GTCTACGTATGCAAGCACCACATCTTAA
**Non-complementary target**	CTAGATCCGTGTCCTCGTGGCCGC

S-III Oligonucleotide synthesis and purification

Oligonucleotides were synthesized on an Applied Biosystems 394 automated DNA/RNA synthesizer using a standard 1.0 μmole phosphoramidite cycle of acid-catalyzed detritylation, coupling, capping, and iodine oxidation. Stepwise coupling efficiencies and overall yields were determined by the automated trityl cation conductivity monitoring facility and were >98.0%. All β-cyanoethyl phosphoramidite monomers were dissolved in anhydrous acetonitrile to a concentration of 0.1 M immediately prior to use with coupling time of 50 s for normal A, G, C, and T monomers and was extended to 600 s for 5'-Amine monomer (5'-TFA-Amino-Modifier C6-CE Phosphoramidite was purchased from Link Technologies Ltd.).

5'-Amino modified oligonucleotides on resin were treated with a solution of diethylamine (10% in acetonitryl) for 20 min in order to selectively remove the cyanoethyl protecting groups. Cleavage and deprotection of oligonucleotides were achieved by exposure to concentrated aqueous ammonia solution for 60 min at room temperature followed by heating in a sealed tube for 5 h at 55 °C.

Purification was carried out by reversed-phase HPLC on a Gilson system using a Brownlee Aquapore column (C8, 8 mm x 250 mm, 300Å pore) with a gradient of acetonitrile in triethylammonium bicarbonate (TEAB) increasing from 0% to 50% buffer B over 20 min with a flow rate of 4 mL/min (buffer A: 0.1 M triethylammonium bicarbonate, pH 7.0, buffer B: 0.1 M triethylammonium bicarbonate, pH 7.0 with 50% acetonitrile). Elution was monitored by ultraviolet absorption at 298 nm. After HPLC purification, oligonucleotides were freeze dried then dissolved in water without the need for desalting.

All Purified oligonucleotides were characterised by electrospray mass spectrometry. Mass spectra of oligonucleotides were recorded using a XEVO G2-QTOF MS instrument in ES- mode. Data were processed using MaxEnt and in all cases confirmed the integrity of the sequences.

S-IV UV-Vis characterization of GO

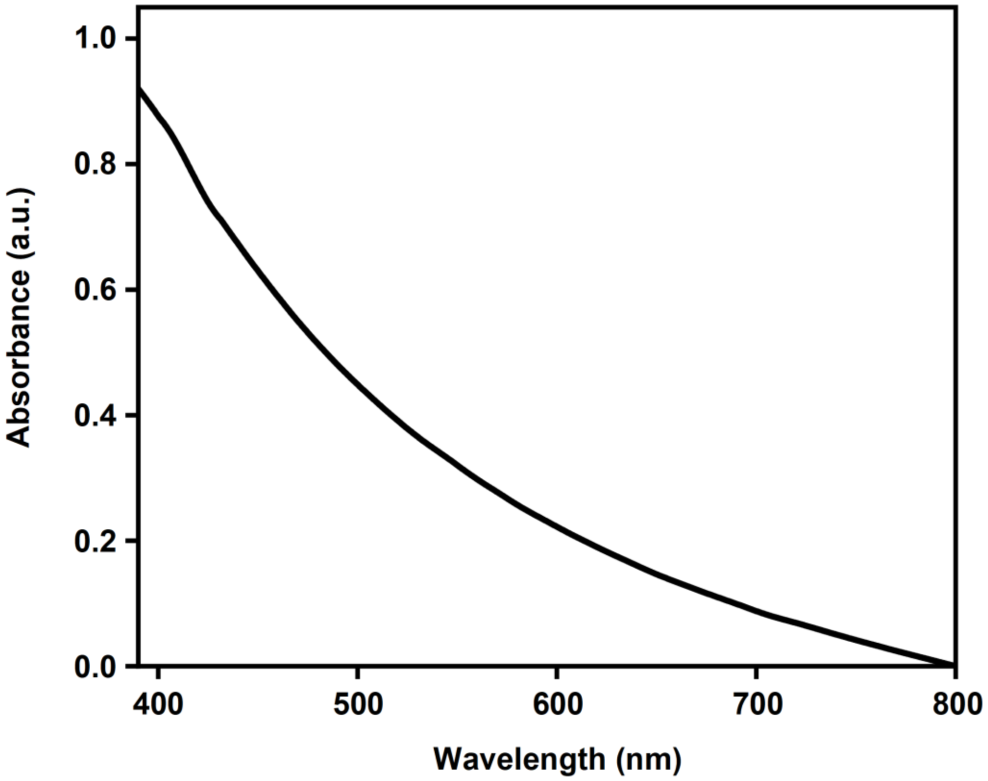

Figure S4. UV-Vis spectrum of GO demonstrating the increased absorbance and 540 nm compared to a wavelength of 655 nm.

S-V Target recognition in biological fluid

After demonstrating the successful response of our sensor to varying concentrations of cDNA we also investigated whether its response would be affected by the presence of biological fluids. **Figure S5** shows the fluorescence signal obtained following incubation of UCNPs, hybridized to cDNA, in saliva, which confirms the function of the sensor.

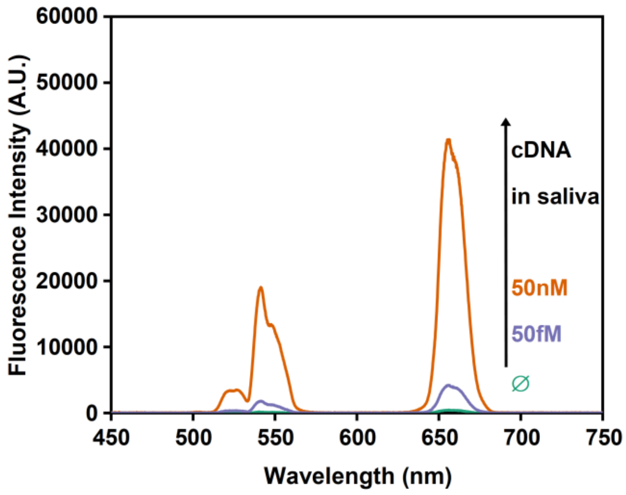

Figure S5. Representative fluorescence spectrum of oligonucleotide coated UNCPs (0.5 mg/mL) after incubation with cDNA in the presence of GO in saliva.

S-VI RNA target recognition

The efficiency of detection of a SARS-CoV-2 oligonucleotide target was tested upon the use of a synthetic cRNA sequence, which was designed to represent the same RdRP/Hel target used through our study (see **Table S1**). **Figure S6** shows that following hybridization of UCNPs with an increasing concentration of cRNA and upon incubation with GO, a gradual increase in fluorescence intensity was observed.

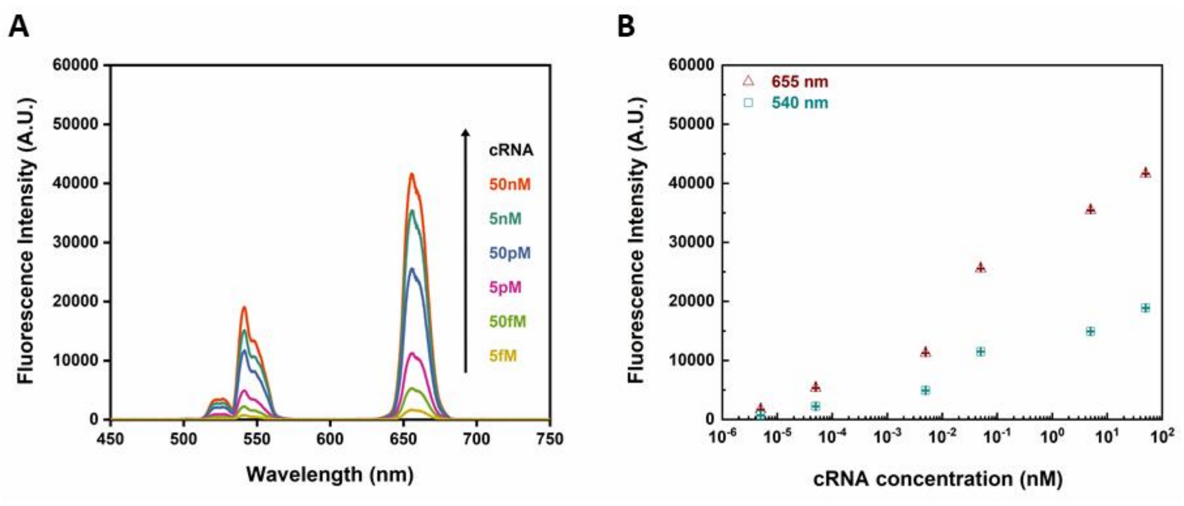

Figure S6. (A) Representative fluorescence spectrum of oligonucleotide coated UNCPs (0.5 mg/mL) after incubation with increasing concentrations of cRNA targets in the presence of GO (B) Graph of the maximum UCNP fluorescence intensity measured at 540 and 655 nm in the presence of GO as a function of cRNA concentration.

S-VII Additional experimental information

Materials

All chemicals were used as received without further purification and were obtained from commercial sources. Yttrium(III) chloride hexahydrate (99.9%), ytterbium(III) chloride hexahydrate (99.9%), erbium(III) chloride hexahydrate (99.9%), ammonium fluoride (98%), 1-octadecene (90%), oleic acid (90%), methanol (99%), chloroform (99.8%), n-hexane (95+%), tetrahydrofuran (99.8%), boric acid (99.99%), 1-(3-dimethylaminopropyl)-3-ethylcarbodiimide hydrochloride (EDC) (98+%) and N-hydroxysulfosuccinimide sodium salt (sulfo-NHS) (97%) were purchased from Alfa Aesar. Sodium hydroxide (97%) and poly(acrylic acid) (PAA) (MW ≈ 1.8 kDa) were purchased from Sigma-Aldrich. Phosphate buffered saline tablets and ethanol (≥99%) were purchased from Thermo Fisher Scientific. GO (black powder of monolayer sheets dispersed in phosphate buffered saline before use) was purchased from Graphene Supermarket. GO monolayers exhibit a flake size between 0.2–2 µm with a monolayer ratio of at least 80%.

Methods

The morphology of UCNPs was analyzed using transmission electron microscopy. The samples were prepared by putting a drop of a diluted nanoparticle solution on a 400 mesh formvar coated copper grid and left to dry. A Hitachi HT7700 Transmission Electron Microscope operating at an accelerating voltage of 100 kV was used. The size analysis was carried out by counting over 200 nanoparticles with ImageJ software (National Institutes of Health, USA). ζ-potential measurements were performed using a Zetasizer Nano ZS instrument (Malvern Instruments, UK) at room temperature and the acquired data were processed using the software provided by Malvern (Zetasizer software v7.13). A Nicolet iS5 FT-IR Spectrometer (Thermo Fisher) was used for Fourier-transform infrared spectroscopy

measurements. Each measurement was collected in the spectral range of 525 and 4000 cm^{-1} at room temperature. The UCNP fluorescence measurements were performed using a 300 mW, 980 nm diode laser (Thorlabs LTD, UK) as an excitation source. A short pass IR-blocking filter (Schott KG3) was part of the setup to suppress scattered excitation light and select only the fluorescence emission. A detector SpectraSuite Spectrometer (OceanOptics, USA) was used to collect the emitted fluorescence perpendicular to the excitation beam using a 35 mm focal length lens. A cuvette filled with the appropriate solvent was illuminated with the 980 nm diode laser beam and measured as the blank. All measurements were performed under 100 ms of integration time and 10 scans to average. The fluorescent experiments were repeated three times and the fitted data corresponded to the mean value ± standard error of the mean.

Synthesis of Core β-$NaYF_4$: Yb, Er UCNPs.

The synthesis of core UCNPs was achieved by following a slightly modified version of a previously reported protocol.[27] Briefly, $YCl_3.6H_2O$ (236 mg, 0.78 mmol), $YbCl_3 \cdot 6H_2O$ (77.5 mg, 0.20 mmol) and $ErCl_3 \cdot 6H_2O$ (7.63 mg, 0.02 mmol) were mixed together with 6 mL of OA and 15 mL of 1-octadecene in a 3-neck round bottom flask. By employing a Schlenk line, the mixture was gradually heated to 150 °C, under Argon flow, for 1h 30 min. The reaction mixture was then cooled to room temperature and a mixture of NaOH (100 mg, 2.5 mmol) and NH_4F (148.16 mg, 4 mmol) dissolved in 10 mL dry methanol was injected dropwise and left to stir at room temperature for a further 45 min. After that, the methanol was evaporated from the mixture for 30 min under Ar at 100 °C followed by another 30 min under vacuum. The reaction mixture was then heated to 310 °C, at a rate of 15°C/min under Ar, for 1h 20 min to form the nanoparticles. Finally, the reaction was left to cool down to room temperature. The purification of the core UCNPs was done by a series of three centrifugations (8000 rpm, 15 min). In between each centrifugation, the pellet was resuspended in EtOH (15mL) *via* a sonication bath. After

the final round of purification, the white UNCP pellet was left to dry at 80 °C before their use further experiments.

Synthesis of Core-Shell UCNPs

Core-shell UCNPs were prepared following a modified version of a previously published protocol.[28] By using a Schlenk line, $YCl_3 \cdot 6H_2O$ (151.68 mg, 0.5 mmol) was dissolved in a mixture of 1-octadecene (15 mL) and OA (6 mL) *via* stirring for 1h under Ar at 150 °C. Then, the reaction mixture was cooled down to 80 °C and the previously synthesised core UCNPs (125 mg) in CH_3Cl (20 mgmL^{-1}) were added dropwise using a syringe. After 20 min at 80 °C, the solution was gradually heated up to 100 °C under Ar for a further 45 min to fully evaporate the CH_3Cl. The reaction mixture was then cooled down to room temperature and a solution of NaOH (50 mg, 1.25 mmol) and NH_4F (74.08 mg, 2 mmol) dissolved in dry MeOH (5 mL) was added dropwise under vigorous stirring. The reaction mixture stirred for a further 45 min at room temperature before being gradually heated to 100 °C under Ar where it was left for 30 min. To ensure full evaporation of MeOH, the reaction was put under vacuum for 30 min. Then the reaction mixture was heated to 310 °C at a rate of 15 °C/min under Ar and left for 1h 40 min for the formation of core-shell UCNPs. After this, the reaction was left to cool down to room temperature before purification (three times centrifugation at 8000 rpm for 15 min and washing with EtOH). The core-shell UCNPs' pellet was collected and re-dispersed in tetrahydrofuran.

Ligand exchange with (Poly)acrylic acid (PAA)

The UCNPs surface was further functionalized in a homogeneous ligand exchange step with PAA to bring the UCNPs in water. Briefly, a solution of PAA (250 mg, MW ≈ 1.8 kDa) dissolved in tetrahydrofuran (3 mL) was added to the core-shell UCNPs coated with OA (21 mg in 7 mL tetrahydrofuran). To allow for ligand exchange, the mixture was stirred for 96 h at

room temperature. After the reaction, the nanoparticles were collected *via* centrifugation and washed with ethanol (20 mL) twice. The particles' pellet was dried and re-suspended in phosphate buffered saline and stored at 4 °C.

Synthesis and characterisations of oligonucleotide coated UCNPs

The PAA coated core-shell UCNPs were incubated with the amino-modified oligonucleotides and functionalized *via* the carboxylic groups on the PAA ligand using EDC amino-coupling chemistry. A solution of EDC (20 µL, 0.3 M) and sulfo-NHS (40 µL, 0.3 M) in 4-morpholineethanesulfonic acid buffer (pH 5.5, 0.1 M) was added to PAA coated core-shell UCNPs (0.5 mg/mL) suspended in borate buffer (pH 8.5, 0.01 M). After sonicating the mixture for 10 min, the desired amino-terminated oligonucleotide sequence was added (9 nmol). The solution was left under stirring overnight. The particles were then purified by centrifugation three times. The oligonucleotide UCNPs were resuspended in sterile DNAse/RNAse free Milli-Q water and stored at 4 °C.

Sensor calibration

A stock solution of 5 mg/mL GO is prepared by dissolving 50 mg of GO in 10 mL phosphate buffered saline. The calibration was carried out by adding increasing concentrations of GO, in 0.1 mg/mL increments to a dispersion of a fixed concentration of oligonucleotide coated UCNPs (0.5 mg/mL). The corresponding fluorescence spectra of the UCNPs were monitored to determine the concentration of the GO that would result in optimum fluorescence quenching.

Targeted DNA/RNA detection using oligonucleotide coated UCNPs

The oligonucleotides on the UCNP surface were hybridized with their complementary sequence before incubating with the GO to prevent the interaction between the single-stranded DNA coated UCNPs and the GO. 0.5 mg/mL of oligonucleotide coated UCNPs were incubated in phosphate buffered saline with various concentrations of the cDNA strand (ranging from 5

fM to 5 nM) while shaking. Then, a solution of GO dispersed in phosphate buffered saline was added and left incubating for 10 min before performing the fluorescence measurements.
